# Retrotransposon gag-like 1 (RTL1) and the molecular evolution of self-targeting imprinted microRNAs

**DOI:** 10.1186/s13062-019-0250-0

**Published:** 2019-10-22

**Authors:** Avantika Mainieri, David Haig

**Affiliations:** Department of Organismic and Evolutionary Biology, Harvard University, Cambridge, MA USA

**Keywords:** Genomic imprinting, Greenbeard effect, Protein tandem repeats, Chloropogonology

## Abstract

**Background:**

Transcription of the antisense strand of *RTL1* produces a sense mRNA that is targeted for degradation by antisense microRNAs transcribed from the sense strand. Translation of the mRNA produces a retrotransposon-derived protein that is implicated in placental development. The sense and antisense transcripts are oppositely imprinted: sense mRNAs are expressed from the paternally-derived chromosome, antisense microRNAs from the maternally-derived chromosome.

**Results:**

Two microRNAs at the *RTL1* locus, miR-431 and the rodent-specific miR-434, are derived from within tandem repeats. We present an evolutionary model for the establishment of a new self-targeting microRNA derived from within a tandem repeat that inhibits production of RTL1 protein when maternally-derived in heterozygotes but not when paternally-derived.

**Conclusions:**

The interaction of sense and antisense transcripts can be interpreted as a form of communication between maternally-derived and paternally-derived *RTL1* alleles that possesses many of the features of a greenbeard effect. This interaction is evolutionary stable, unlike a typical greenbeard effect, because of the necessary complementarity between microRNAs and mRNA transcribed from opposite strands of the same double helix. We conjecture that microRNAs and mRNA cooperate to reduce demands on mothers when an allele is paired with itself in homozygous offspring.

**Reviewers:**

This article was reviewed by Eugene Berezikov and Bernard Crespi.

## Background

From his experiments on hybridization in peas, Gregor Mendel concluded that “it is perfectly immaterial whether the dominant character belong to the seed-bearer or to the pollen-bearer; the form of the hybrid remains identical in both cases” [1]. This identity of reciprocal heterozygotes was a largely unquestioned assumption of Mendelian genetics until the discovery of ‘imprinted’ loci at which alleles were differentially expressed depending on whether they were inherited via an egg or a sperm [2]. The *callipyge* (*CLPG*) phenotype of sheep (hypertrophied muscles of the ovine rump) violates basic Mendelian presumptions. Sheep-breeders were unable to fix this desirable trait within a herd because the only animals to express the trait inherited the underlying mutation from their father but not their mother (+/*CLPG*). The reciprocal heterozygotes who inherited the mutation from their mother (*CLPG*/+) had rumps of normal proportions as did sheep who inherited the mutation from both parents (*CLPG*/*CLPG*) or neither parent (+/+) [3]. The difference between the heterozygous genotypes could be explained by genomic imprinting but the normal phenotype of both homozygous genotypes implied some unknown interaction between maternal and paternal chromosomes.

The *callipyge* region of ovine chromosome 18 and orthologous regions of human chromosome 14 and mouse chromosome 12 were subsequently found to be the home of a major cluster of imprinted genes [4–6] including many non-coding RNAs [7–9]. One gene from this cluster, *Retrotransposon Gag like 1* (*RTL1*), is derived from a *sushi-ishi*-like retroelement [10, 11] that has retained a protein-coding sequence in eutherian but not marsupial genomes [12]. RTL1 protein is encoded by a single exon, with conservation of the capsid and protease domains of the ancestral retroelement, but with loss of critical residues in polymerase, RNaseH and integrase domains [10]. The paternal copy of *RTL1* is transcribed from the antisense strand of the DNA double helix to produce a protein-coding ‘sense’ mRNA whereas the maternal copy is transcribed from the sense strand to produce multiple ‘antisense’ microRNAs [13, 14]. In some cells, these microRNAs are coordinately transcribed as part of a long antisense transcript [14]. In other cells, individual microRNAs are transcribed from unique promoters [15, 16]. These microRNAs are complementary to the *RTL1* coding sequence and promote degradation of *RTL1* mRNA [17–19]. The *RTL1* sequence is thus constrained by dual roles as the progenitor of a paternally-expressed mRNA and maternally-expressed microRNAs.

*Rtl1* mRNA is expressed in endothelial cells of the labyrinthine zone of the murine placenta. Deletion of the coding sequence results in complete suppression of *Rtl1* mRNA when inherited from fathers (paternal-knockout) but three-fold increased *Rtl1* mRNA when inherited from mothers (maternal-knockout). The latter effect is a consequence of the absence of the maternally-expressed antisense microRNAs that target paternally-expressed *Rtl1* mRNA [20]. Both knockouts are associated with abnormal placental morphology. Mice with the paternal knockout suffer prenatal growth retardation associated with detachment of endothelial cells from the basement membrane of placental capillaries [21]. Mice with the maternal knockout have normal birth weight but suffer post-weaning growth retardation.

Overexpression of *RTL1* causes muscular hypertrophy in mice, suggesting a role of the gene in the *callipyge* phenotype of sheep [22], induces hepatocellular carcinomas in mice [23], and promotes cellular proliferation in human melanoma [24]. Non-expression of *RTL1* is the major cause of post-implantation failure of cloned pigs [25]. A critical evaluation of all the reported effects of *RTL1*-associated miRNAs, both pro-oncogenic and anti-oncogenic, is beyond the scope of this paper.

### Genomic imprinting and intragenomic conflict

The kinship theory of genomic imprinting was developed to explain the origin and maintenance of imprinted gene expression in prenatal and postnatal life [26, 27]. Simple models predicted that incremental decreases in the expression of paternally-expressed genes (PEGs) would reduce patrilineal fitness whereas incremental decreases in the expression of maternally-expressed genes (MEGs) would reduce matrilineal fitness [26]. In these models, fitnesses were a function of the level of gene product *X* = *x*_m_ + *x*_p_, where *x*_m_ was the level of expression of the maternally-derived allele and *x*_p_ the level of expression of the paternally-derived allele. An allele’s ‘strategy’ in these two roles was represented by the two-element vector {*x*_m_, *x*_p_}. The models found maternal silence {0, *X*} to be the unbeatable strategy at loci where higher values of *X* were favored when paternally-derived and paternal silence {*X*, 0} to be the unbeatable strategy at loci where higher values of *X* are favored when maternally-derived.

In the context of maternal–fetal relations and placental development, the theory predicts that PEGs will increase fetal demands on mothers and MEGs will have opposing effects [28]. The classic example is the opposite effects on embryonic growth in mice of *Insulin-like growth factor 2* (*Igf2*), a paternally-expressed growth-enhancer, and *Insulin-like growth factor 2 receptor* (*Igf2r*), a maternally-expressed growth-inhibitor that targets IGF-II to lysosomes for degradation [29]. A single amino-acid substitution in the IGF2-binding site of the IGF2R protein resulted in embryonic and placental overgrowth in mice because it abolished clearance of IGF2 by the receptor [30]. Similar antagonistic effects on fetal growth are observed in humans. Fetal overgrowth can be caused by mutational inactivation of the maternal copy of *CDKN1C*, a paternally-silent growth-inhibitor [31] whereas intrauterine growth-restriction can be caused by mutational inactivation of the paternal copy of *IGF2*, the aforementioned maternally-silent growth-enhancer [32].

The opposite effects of paternally-expressed *RTL1* mRNA and maternally-expressed microRNAs on levels of RTL1 protein have been interpreted as consistent with the kinship theory [33]. Moreover, the prenatal growth retardation observed in paternal knockouts of *Rtl1* is compatible with RTL1 protein functioning as a fetal demand enhancer and the increase of *Rtl1* mRNA when the imprinted microRNAs are knocked-out is compatible with the microRNAs acting as demand inhibitors [20, 21]. These hypotheses remain to be tested in experimental systems that cause less severe perturbation in the level of *Rtl1* mRNA.

The conclusion that the phenotypic effects of *RTL1* support the kinship theory is premature because the details of this system violate a key assumption of the mathematical models. This was the assumption that *x*_m_ and *x*_p_ were fixed properties of an allele and therefore independent of the identity of the other allele at the locus in a diploid individual. An *RTL1* allele is transcribed both as a PEG (mRNA) and as MEGs (microRNAs) that inhibit the PEG. Therefore, the level of expression of the paternal allele may depend on the identity of the maternal allele. At such a locus, an allele’s strategy could be thought of as a three-element vector {*x*_m_, *x*_p_, *x*_q_} where *x*_m_ and *x*_p_ are the levels of expression when maternally-derived and paternally-derived in heterozygotes, and *x*_q_ is the level of expression in homozygotes.

Haig [34, 35] made a first attempt to address this theoretical puzzle in the context of microRNAs expressed during seed development. He argued that imprinted noncoding RNAs are subject to distinctive selective forces when they regulate transcripts of the allele inherited from the other parent. If an mRNA possesses a sequence complementary to the imprinted noncoding RNA, and the complementary sequences pair, then the mRNA can be considered to ‘recognize’ its similarity to the allele from which the microRNA was transcribed and can evolve to respond strategically to this information. By this means, the maternal and paternal alleles at a locus could coordinate their activities. The noncoding RNA functions as a signal by which one allele ‘communicates’ its presence to the other. Such strategic coordination between alleles would possess the formal properties of a ‘greenbeard effect’ with possibilities for ‘altruistic’ behavior in homozygotes but ‘selfish’ behavior in heterozygotes [35].

Classic formulations of greenbeard effects invoked a triad of trait, recognition, and response: an actor with the trait recognized the same trait in another individual and responded altruistically [36, 37]. Such effects were dismissed as implausible because it was considered unlikely that the three aspects of the triad could be instantiated at a single locus. Interest in greenbeards has revived because of the realization that recognition of identity at the molecular level is simpler than recognition of relatedness [38] and because theoretical models find that tight linkage of trait, recognition, and response is not necessary to facilitate the evolution of cooperation [39].

A microRNA can be considered a *trait* that is *recognized* by the microRNA’s target and that *informs* the targeted sequence of its similarity to the sequence from which the microRNA was transcribed. Regulation of the targeted sequence by the microRNA can be considered the *response* of the target to this information. If the microRNA acts on an mRNA transcribed from the other allele at its locus, then regulation of the mRNA by the microRNA can be conceptualized as a response to self-recognition. ‘Self-targeting’ microRNAs thus combine all three aspects of the greenbeard triad in a single nucleic acid sequence.

The mammalian *RTL1* locus meets these criteria for ‘self-recognition’ and thus provides a test-case for disentangling the evolutionary complexities of such systems. The current paper investigates the evolutionary history of microRNAs at the *RTL1* locus and then develops an evolutionary model of how some features of this complex system could have evolved.

## Results

### mRNAs processed from *RTL1-antisense* transcripts

**Fig. 1:**
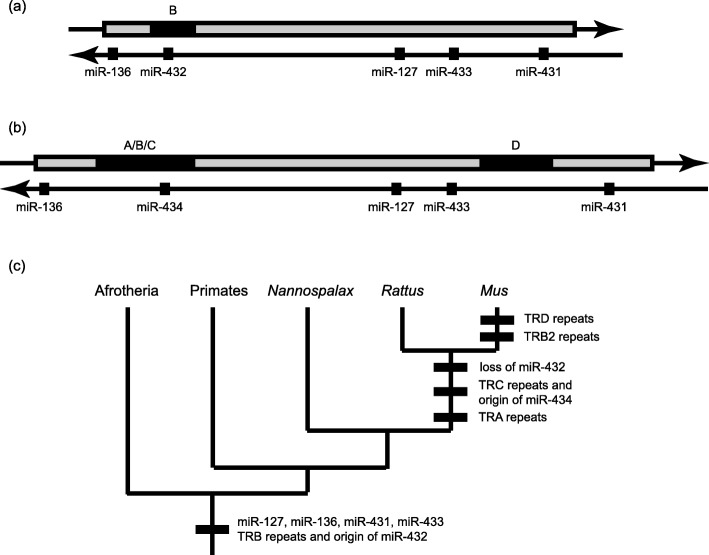
The location of tandem repeats (**A**–**D**) within the protein-coding region of the sense strand (above) and of microRNAs on the antisense strand (below) of (**a**) human *RTL1* and (**b**) mouse *Rtl1*. (**c**) Phylogenetic ‘road-map’ of when particular features arose

Our results build upon prior analyses of the *RTL1* sequence [10, 18]. We obtained *RTL1* sequences from the reference genomes of representative primates (*Homo*, *Callithrix*), rodents (*Mus*, *Rattus*, *Nannospalax*, *Heterocephalus*, *Cavia*), afrotherians (*Elephantulus*, *Orycteropus*, *Trichechus*) and xenarthrans (*Dasypus*, *Choloepus*). Figure 1 will be useful for orientation in the discussion of our results. It shows the relative locations of microRNAs and tandem repeats in the human (Fig. 1a) and mouse *RTL1* genes (Fig. 1b) and provides an overview of when particular features arose (Fig. 1c).

Sequences highly similar to pre-miR-136, pre-miR-127, and pre-miR-433 were found in all species. Sequences highly similar to pre-miR-431 were found in all species except xenarthrans. We do not know whether this is a real or artifactual absence because of gaps in xenarthran genome assemblies. We also found sequences similar to pre-miR-432 in all species although these sequences had undergone substantial divergence in rats and mice with loss of functional miR-432. Therefore, pre-miR-432 can be inferred to have been present in an ancestor of all extant eutherian mammals but to have been lost within the rodent lineage where it has been replaced by miR-434. Alignments of these sequences are provided in Additional file 1: Figure S1.

Our analysis will distinguish between ‘autotargets’ and ‘allotargets’. An autotarget is a sequence that is directly complementary to a microRNA because it is transcribed from the opposite strand of the DNA double helix. Allotargets are sequences that are complementary to the microRNA’s seed but are not autotargets. The distinction between autotargets and allotargets is evolutionarily significant because changes to seed sequences are necessarily associated with corresponding changes to autotargets because microRNA and autotarget are transcribed from opposite strands of the *RTL1* DNA double helix. By contrast, changes to seed sequences are not associated with complementary changes to allotargets. (The seed is the 7-nucleotide sequence principally responsible for functional binding of microRNAs to their targets.)

**Fig. 2:**
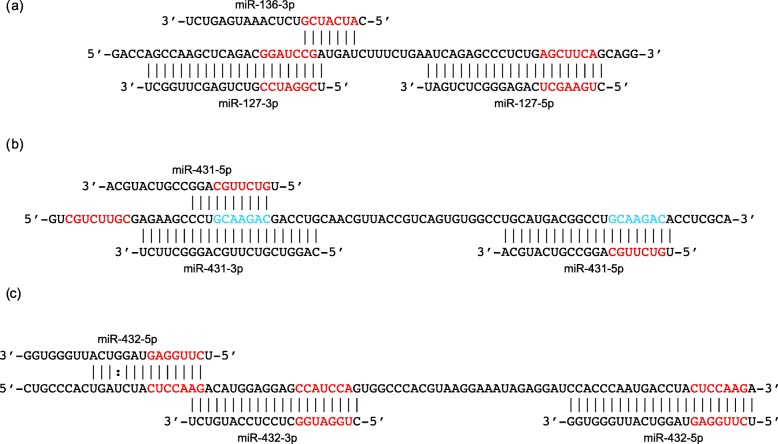
Antisense microRNAs with allotargets on human *RTL1* sense mRNA: (**a**) an allotarget of miR-136-3p overlaps the autotarget of miR-127-3p; (**b**) an allotarget of miR-431-5p overlaps the autotarget of miR-431-3p; (**c**) an allotarget of miR-432-5p overlaps the autotarget of miR-432-3p

Three of the antisense microRNAs have predicted allotargets on the sense mRNA (Fig. 2): miR-136-3p has a highly-conserved allotarget that overlaps the autotarget of miR-127-3p; miR-431-5p has a conserved allotarget overlapping the autotarget of miR-431-3p; miR-432-5p has a conserved allotarget overlapping the autotarget of miR-432-3p. In addition to targets on the sense mRNA, at least four microRNAs (miR-127-3p, miR-431-5p, miR-432-5p, miR-433-3p) have predicted allotargets on the antisense pre-miRNA sequences from which they are processed. Of particular interest, nucleotides 2–9 of miR-127-3p (CGGAUCCG) are self-complementary as are nucleotides 1–8 of miR-433-3p (AUCAUGAU). Thus, these microRNAs possibly bind to their own seed sequences (Additional file 1: Figure S2).

Our in silico predictions of intralocus allotargets hint at hidden complexities of RNA–RNA interactions (and possibly RNA–DNA interactions) at the *RTL1* locus. We acknowledge the limitations of non-experimental analyses but hope that they can provide some guidance for experiment. Experimental data on the interaction between mRNA and microRNAs at the *RTL1* locus are currently limited. The microRNAs are known to initiate cutting of *RTL1* mRNA but the specific sequences targeted by each microRNA are unknown. We identified potential targets by the criterion of perfect matches to seed sequences, but complementarity is not proof that an interaction occurs in vivo and may miss targets that lack perfect complementarity to the seed sequence (so-called non-canonical targets). Is the cleavage of *RTL1* mRNA by a microRNA mediated by its binding to its autotarget that is complementary to the entire length of the microRNA or by its binding to allotargets that are complementary only to the seed sequence?

### Tandem repeats at the *RTL1* locus

A notable feature of prior analyses of the *RTL1* sequence [10, 18] was the presence of tandem repeats within the protein-coding sequence named TRA, TRB, TRC, and TRD by Davis et al. [18].

Thirty-three nucleotides separate the initial nucleotides of miR-432-5p’s allotarget and autotarget on the *RTL1* mRNA. This 33-nucleotide periodicity corresponds to one TRB unit. miR-432 is processed from within the complementary TRB array of antisense transcripts. We detected seven degenerate copies of TRB in the *RTL1* genes of afrotherians, xenarthrans, humans and non-muroid rodents (Additional file 1: Figure S3). We therefore conclude that these repeats originated before the divergence of all extant eutherian mammals and that at least three TRBs were present at the origin of miR-432 because pre-miR-432 extends across more than two repeats. TRB repeats are older than the other repeats discussed below and the individual units have undergone greater evolutionary divergence.

The rat and mouse *Rtl1* genes contain 12-nucleotide TRA repeats located immediately adjacent to the ancestral TRB repeats. We count ten TRA units in mouse *Rtl1* and at least twenty in rat *Rtl1*. The rat and mouse *Rtl1* genes also contain 24-nucleotide TRC repeats that expanded from within the TRB repeats. These repeats are located immediately 5′ of the sequence complementary to pre-miR-432 on the sense strand (Additional file 1: Fig. S4). We count twelve TRC units in mouse *Rtl1* and twenty-three TRC units in rat *Rtl*. The TRA and TRC repeats are absent from the *Rtl1* genes of non-muroid rodents. miR-434 is processed from within the TRC arrays of rats and mice. Because of the duplication of TRC units, mouse *Rtl1* possesses four perfect matches (GGUUCAA) to the miR-434-3p seed, three of them allotargets, and three perfect matches (AGUCGAG) to the miR-434-5p seed, two of them allotargets (Additional file 1: Fig. S5). miR-434-5p and miR-434-3p both degrade *Rtl1* mRNA in mice [18].

A 66-nucleotide sequence within the ancestral pre-miR-432 sequence has undergone six-fold tandem amplification in the *Rtl1* gene of mice (we will call these TRB2 units because each contains two TRB units). As a consequence, the mouse *Rtl1* mRNA (sense) contains seven perfect matches (CCAUCCA) to the seed of miR-432-3p and two perfect matches (CUCCAAG) to the seed of miR-432-3p. However, the TRB2 repeats disrupt formation of the pre-miR-432 hairpin. The rat *Rtl1* gene also cannot produce pre-miR-432 but, in this case, because of deletions within the ancestral pre-miR-432 sequence. Deep-sequencing reported in miRBase detects miR-432 in neither rats nor mice [40]. We have not resolved the relative timing of the loss of miR-432 and gain of miR-434.

TRB and TRC repeats exhibit interesting parallels (Additional file 1: Figure S6). TRB and TRC units are ‘partial palindromes’ with internal self-complementarity. Tandem duplications of the repeat units created larger palindromes that fold back upon themselves to form the hairpins from which pre-mir-432 and pre-miR-434 are processed. Both arrays contain more units than are required to generate the embedded pre-miRNAs. One model of the origin of pre-miRNA hairpins derives them from inverted duplications [41]. The tandem duplications that generated pre-mir-432 and pre-miR-434 do not fit this model but in some respects resemble the alternative model in which partial self-complementarity of the ascending and descending arms of pre-miRNA hairpins arises by ‘chance’ [42]. However, in the case of pre-mir-432 and pre-miR-434, the length of self-complementarity generated by chance was greatly reduced because of tandem duplication of already self-complementary units.

Finally, the mouse *Rtl1* sequence contains an AG-rich region of 33-nucleotide repeats (TRDs) that is remarkable for a stretch of 442 nucleotides without a cytosine (227 guanines, 192 adenines, 23 uracils). At the amino acid level, this region encodes 115 glutamic acids, 14 aspartic acids, and 14 glycines. TRD repeats are absent from the *Rtl1* genes of rats and non-rodents.

The lengths of the various repeat-units are all multiples of three—TRA (12), TRB (33), TRB2 (66), TRC (24), TRD (33)—as required to preserve the open reading frame of *RTL1* mRNA. An intriguing question is whether the principal selective forces in the fixation of these repeats acted on properties of the RNA sequence or properties of the encoded amino acids. Many proteins contain internal repeats with a variety of functions [43] but we conjecture that the complex repeated structures associated with miR-432 and miR-434 (TRA, TRB, TRB2, TRC) were primarily shaped by interactions between sense and antisense RNAs rather than by functional properties of the RTL1 amino-acid sequence.

The TRD repeats of mouse *Rtl1* are not associated with a microRNA. It is possible that these repeats evolved because of their contribution to the amino acid sequence of Rtl1 protein. Another possibility is that the repeats are associated with formation of an R-loop between the C-rich template DNA and G-rich RNA transcript. In this configuration, the G-rich non-template DNA might fold back upon itself to form G-quartet structures [44]. Such an R-loop would impede transcription of sense *Rtl1* mRNA.

### *cis* and *trans* effects

The establishment of imprints in mammalian genomes involves interactions between *trans*-acting and *cis*-acting factors in parental germlines: an imprinting control region (ICR) is differentially marked in male and female germlines by *trans*-acting factors, then the epigenetically-inherited state of the ICR determines expression of imprinted genes in *cis* in the next generation [2]. The distinction between *trans*-acting and *cis*-acting factors is crucial for understanding the evolution of genomic imprinting in mammals. If there is heterozygosity at a *trans*-acting locus that acts in the mother’s germline, an allele can influence gene expression in all of a mother’s offspring, not just the 50% that receive the *trans*-acting allele. By contrast, if there is heterozygosity at a *cis*-acting locus, then the effect is experienced only by those offspring that inherit the *cis*-acting allele. Therefore, a gene that acts in *trans* to imprint an ICR is subject to different selective forces than the ICR that acts in *cis* to determine gene expression in the next generation. This is a source of evolutionary conflict between maternal genes (in mothers) and maternally-derived genes (in offspring) [45] and has been conceptualized as a conflict between imprinting genes and imprinted genes [46].

Conflict between imprinting genes and imprinted genes occurs because of their independent segregation at meiosis. Conflict is not predicted when *trans*-acting loci modify *cis*-acting loci in the haploid phase because *trans*-acting and *cis*-acting alleles are necessarily inherited together in products of syngamy. The establishment of epigenetic marks in gametophytes (haploid phase of plants) rather than sporophytes (diploid phase of plants) may explain why ‘imprinting’ genes are imprinted in plants, but not mammals, and why clusters of imprinted genes under *cis* control of an ICR have not been described in plants [34]. Conflict is also not predicted between *trans*-acting and *cis*-acting loci whose effects are restricted to the diploid generation in which the loci act.

### A model of the origin of self-targeting microRNAs

Genetic effects in which one allele influences the expression of its homolog have been labelled *cum* effects [34] or *trans*-homolog interactions [17]. Such effects, as observed at the *RTL1* locus, do not map neatly onto the *cis* versus *trans* distinction: they occur at the same locus but not on the same chromosome. In this section, we present a model in which self-targeting microRNAs are derived from tandem repeats. The model illustrates how *cum* effects are subject to distinctive selective forces from *cis* and *trans* effects. The model was inspired by the observation that miR-432 and miR-434 are derived from tandemly-repeated sequences in the *RTL1* gene.

**Fig. 3:**
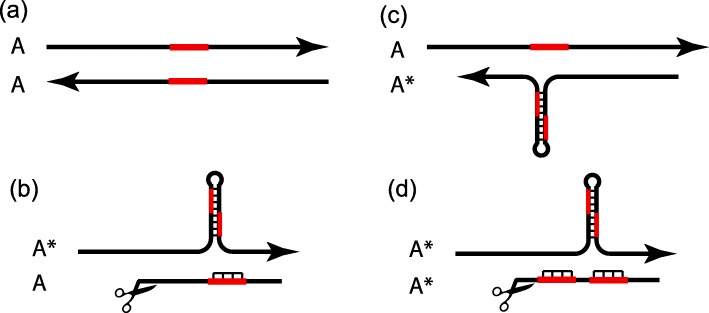
At the *RTL1* locus an ancestral *A* allele has a single copy of a partially palindromic sequence (indicated in red) and the derived A* allele has a tandem duplication of this sequence which is processed as a microRNA from the maternal antisense transcript. **a** In *AA* homozygotes the maternal allele (above) is expressed as *RTL1-antisense* noncoding RNA and the paternal allele (below) is expressed as sense *RTL* mRNA. No microRNAs inhibit translation of *RTL1* mRNA. **b** In A*A heterozygotes, the maternal A* allele produces a microRNA that binds to the mRNA transcribed from the paternal *A* allele which possesses a single-copy of the partially palindromic sequence. Translation of RTL1 protein is inhibited. **c** In AA* heterozygotes, the *A* maternal allele does not produce the microRNA and the paternal A* mRNA is translated. **d** In A*A* homozygotes, the maternal A* allele is processed as a microRNA that binds to both copies of the palindromic sequence of the mRNA transcribed from the paternal A* allele. Translation of RTL1 protein is inhibited

Consider an ancestral allele (*A*) of *RTL1* that contains a single copy of a partially palindromic sequence. In *AA* homozygotes, the maternal allele is expressed as an antisense non-coding RNA and the paternal allele is expressed as a sense *RTL1* mRNA (Fig. 3a). Now consider the introduction into a population fixed for *A* of a new allele A* with a tandem duplication of the palindromic sequence that forms a hairpin from which an antisense microRNA is processed. In an outbred population, A* is initially present only in A*A heterozygotes who inherit A* from their mother or AA* heterozygotes who inherit A* from their father. In A*A heterozygotes, the maternally-expressed microRNA targets the paternally-expressed mRNA (Fig. 3b). In AA* heterozygotes, the maternally-expressed antisense transcript does not produce a microRNA and therefore does not inhibit translation of RTL1 protein (Fig. 3c). Thus, A* reduces production of RTL1 protein when maternally-inherited, via a *cum* effect, but not when paternally-inherited. If RTL1 protein reduces fetal demand, then the microRNA acts as a maternally-expressed demand inhibitor.

Once A* becomes frequent in the gene pool, it encounters itself in A*A* homozygotes. The maternally-expressed A* microRNA then targets the paternally-expressed A* mRNA reducing translation of RTL1 protein (Fig. 3d). Paternal A* thus possesses the ‘neat trick’ of decreasing RTL1 protein, and demands on the mother, when the maternal allele is A* but not when it is *A*. A* thereby exploits *A* in AA* heterozygotes but exhibits ‘self-restraint’ in A*A* homozygotes. If A* mRNA detects A* microRNA, then there is an ‘altruistic’ reduction in RTL1 translation. If the A* mRNA does not detect A* microRNA, then ‘selfish’ levels of RTL1 are produced.

Green-beard effects are generally considered evolutionary unstable because of the threat of ‘false beards’: variants that enjoy the benefits of the altruism of other alleles but do not reciprocate when the roles are reversed. In terms of our model, a population fixed for A* could be invaded by a variant A** that continues to inhibit paternal expression of A* in A**A* heterozygotes but evades restraint (or partially evades restraint) in A*A** heterozygotes. Thus, maternal A** would enjoy the benefit of ‘altruistic’ restraint by paternal A*, but paternal A** would act ‘selfishly’ and not reciprocate (or incompletely reciprocate) when the roles were reversed.

If one assumes that increased transcription increases the number of mRNAs that escape degradation, then the simplest example of A** would be an allele with a variant sense promoter that increased transcription of the paternally-expressed mRNA. This would favor an allele (A***) with a variant antisense promoter that increased transcription of the maternally-expressed microRNA. Such considerations predict an escalation in the strength of antagonistic promoters. Another possibility would be the acquisition, by the mRNA, of ‘decoy’ target sites for the microRNA that ‘sponge’ the microRNA but are resistant to cleavage.

The model presented above for the introduction of a single microRNA could potentially explain the origin of multiple self-targeting microRNAs encoded by the *RTL1* sequence because the model is iterable. One could think of this process as successive introductions of new traits for self-recognition: a green beard, followed by a blue mustache, followed by red whiskers. Once a trait is introduced, it should tend to be maintained by natural selection because changes to the target site on paternally-expressed mRNA to evade repression would be associated with changes to the maternally-expressed microRNAs which would thereby be less effective inhibitors of established alleles. This is a simple consequence of the complementarity of targeting and targeted sequences transcribed from opposite strands of the same double helix.

Maternal resources are predicted to be least efficiently distributed when allocation is controlled by paternally-expressed genes in offspring, more efficiently distributed when controlled by maternally-expressed genes in offspring, and most efficiently distributed when controlled by genes expressed in mothers [45]. The level of RTL1 protein is jointly determined by products of the maternal and paternal allele at the *RTL1* locus. We conjecture that coordination between *RTL1* alleles allows greater reproductive efficiency relative to control by a simple paternally-expressed demand enhancer. The cooperation posited here has some resemblances to the ‘coadaptation’ theory of genomic imprinting that posits an advantage from ‘matching’ of expressed alleles between parents and offspring [47, 48], although in our model the relevant ‘matching’ is between the maternal and paternal alleles of offspring.

We acknowledge the limitations of this model. An ideal population-genetic model would describe the dynamics of the system for all allele frequencies. We have found such models to be analytically intractable. It is difficult to model fitness trade-offs within families mediated by offspring demand in a population-genetic framework. Most previous models have avoided these complications by seeking an evolutionary stable strategy (ESS) that cannot be invaded by alternative strategies when an allele is near fixation in the population. We did not use an ESS approach because greenbeard effects are not predicted to be evolutionarily stable.

### Sequence conservation at the *RTL1* locus

Greenbeard altruism has been predicted to be vulnerable to exploitation by mutations that maintain the trait, and thus receive the benefits, but do not respond in kind (so-called ‘false beards’). As a consequence, evolutionary models of greenbeard effects often predict periods of cooperation interspersed with periods of exploitation, accompanied by turnover in the tags used in self-recognition [49]. Despite this prediction, the antisense microRNAs associated with the *RTL1* genomic sequence are, for the most part, highly conserved: pre-miR-136, pre-miR-127, and pre-miR-433 are highly similar in all eutherian *RTL1* genes, as is pre-miR-432 (except in muroid rodents) and pre-miR-433 (except in xenarthrans where the sequence has not been found).

A number of factors may have stabilized *cum* interactions at the *RTL1* locus. First, the greenbeard triad of trait, recognition and response are all instantiated by the same short sequence and its antisense complement. ‘False beards’ may be difficult to evolve because mutational changes to autotargets that allow the paternally-expressed mRNA to evade inhibition by unmutated maternally-expressed microRNAs would come at the cost of a failure of maternally-expressed microRNAs with the mutated seed to inhibit paternally-expressed mRNAs with the unmutated target. Second, escalation in the strength of sense and antisense promoters increases the cost of loss of inhibition. Third, the accumulation of *trans* allotargets creates a selective force that opposes loss of the microRNA. Such allotargets can be considered to have ‘eavesdropped’ on the conversation between *RTL1* sense and antisense transcripts.

Despite evolutionary conservation of the *RTL1* antisense microRNAs in most eutherian mammals, we find evidence of two episodes of rapid evolutionary change in the *RTL1* sequence. First, we infer an early epoch of rapid change before the divergence of the major clades of extant eutherian mammals. During this period, the *RTL1* gene originated from a retroelement, became subject to imprinted expression and antisense transcription, and gave rise to at least four self-targeting microRNAs. We are unable to tell whether these changes comprised a single episode of rapid change or multiple episodes. The details are lost in the fog of evolutionary time.

Second, we infer a relatively recent episode of rapid change in the *Rtl1* genes of muroid rodents. The neighborhood of the ancestral pre-miR-432 has undergone an expansion of TRA and TRC repeats in an ancestor of rats and mice and of TRB2 repeats in the mouse lineage. Moreover, numbers of TRA and TRC repeats differ between mouse and rat *Rtl1* genes suggesting changes in one or both lineages have continued after rats and mice diverged. miR-434 is present, and miR-432 absent, in both rats and mice. We hypothesize that miR-434 functionally replaced miR-432 in self-targeting of *Rtl1* mRNA. This replacement involved the loss of the miR-432 seed sequence and gain of the miR-434 seed. Our analysis has not resolved whether the gain of miR-434 and loss of miR-432 were evolutionarily contemporaneous or whether there was an extended period in which both microRNAs were produced. Thus, we do not know at what stage in the process miR-432-related sequences were deleted in the rat lineage and TRB2 repeats amplified in the mouse lineage. Some of these questions might be resolved by comparative studies of *Rtl1* genes among muroid rodents.

The origins of miR-432 and miR-434 were both associated with tandem duplications of short repeats and, in both cases, more repeats are present than the number required to generate the pre-miRNA hairpin. Furthermore, differences in the number of repeats between mouse and rat *Rtl1* genes show change in the number of TRC repeats continued after the origin of miR-434. One consequence of the amplification of TRC repeats has been the generation of additional allotargets for miR-434-5p and miR-434-3p. An unanswered question is whether these extra targets increase or decrease degradation of *Rtl1* mRNA. Does the possession of multiple binding sites increase the probability that an mRNA will be degraded, or, do the extra allotargets function as ‘sponges’ [50] that increase the proportion of *Rtl1* mRNAs that escape degradation?

Our model of the origin of self-targeting miRNAs from tandem repeats applies directly to the origins of miR-432 and miR-434, the two microRNAs at the *RTL1* locus that are associated with tandem repeats. miR-127, miR-136, miR-431 and miR-433 are not associated with obvious repeats. Future work could explore whether these microRNAs also originated from tandem repeats, now highly degenerate, or originated in some other way.

## Conclusions

Studies of molecular evolution have focused, for the most part, on the coevolution of DNA and proteins. For example, whether a DNA sequence is under selection has been inferred from the ratio of synonymous to nonsynonymous changes in protein-coding sequences. Much of cellular biology has similarly focused on DNA and proteins. Under the traditional model of cellular control, DNA is transcribed as messenger RNAs that are translated as proteins which are the effective actors within cells. Some of these proteins function as transcription factors that bind to DNA promoters and determine where and when genes are expressed as protein-coding mRNAs.

Noncoding RNAs are increasingly recognized as performing important roles in cellular control. Imprinted chromosomal regions, in particular, are home to many noncoding RNAs, including long noncoding RNAs (lncRNAs), microRNAs (miRNAs), and small nucleolar RNAs (snoRNAs) [51]. The one-to-one nature of complementary pairing between nucleic acid sequences gives these interactions both dosage-sensitivity and sequence-specificity. Imprinted gene products are predicted to be dosage-sensitive in their effects because inactivation of one allele by imprinting would have little effect on fitness at a dosage-insensitive locus [26]. Non-coding RNAs may therefore be particularly predisposed to the origin and evolutionary maintenance of imprinted expression.

The sequence-specificity of interactions between non-coding RNAs and their nucleic acid targets promises a strategic richness of ‘clever tricks’ at imprinted loci. These include opportunities for ‘self-recognition’ in which an RNA recognizes the DNA sequence from which it was transcribed or recognizes RNAs transcribed from the other strand of the DNA double helix. *Cum* effects have been reported at multiple imprinted loci in addition to *RTL1* [17, 52–55] but the mechanisms have not been determined. This paper has investigated the evolution of self-targeting microRNAs at the *RTL1* locus. *DLK1*, the neighboring protein coding locus to *RTL1*, is a paternally-expressed gene (PEG) that is targeted in mice by multiple maternally-expressed microRNAs from the callipyge region [56], another cum effect. Mammalian genomes contain many other imprinted miRNAs but, to our knowledge, none have been shown to act in *cum*. Other examples of antisense miRNAs that target sense mRNAs in *cum* have been found in human and mouse genomes [57] but none are known to be imprinted. An evolutionary understanding of imprinted *cum* effects is in its infancy.

## Reviewers comments

### Reviewer 1: Eugene Berezikov

Mainieri and Haig propose an evolutionary model for the emergence of self-targeting microRNAs from tandem repeats based on the analysis of the imprinted *Rtl1* locus. The authors provide a convincing scenario for the establishment of the greenbeard effect for this locus. The manuscript is clearly written and the logic is easy to follow; I do not find specific flaws in the manuscript. I think this work is a substantial contribution towards evolutionary understanding of *trans*-homolog interactions (*cum* effect) at imprinted loci.

Figure 7 [now Fig. 3] would benefit from more detail. Specifically, indicating the partially palindromic sequence, which serves as a miRNA source and its target, will bring more clarity. In the legend for Fig. 7, under (b) ‘single-copy of the palindromic sequence’ should be ‘a partially palindromic sequence’? It is implied that the main function of the miRNAs in the *Rtl1* locus is to target the *Rtl1* mRNA, thus miRNAs acting as a trait in the greenbeard effect and regulation of the target mRNA as a response. The fact that many miRNAs in this locus are conserved suggests that they have other (probably more prominent) functions. Could it be that the greenbeard effect at this locus has evolved further and inverted, i.e. that the primary function of *Rtl1* mRNA is currently to regulate the activity of its miRNAs via a sponge effect? More discussion on this might be interesting.

***Authors’ response***

We thank Dr. Berezikov for his close reading of the manuscript and have made the requested changes in Fig. 3. The possibility of the *Rtl1* mRNA acting as a sponge for the antisense miRNAs is an interesting one. We raised this possibility briefly in our discussion of the amplifications of TRC repeats in rodents. A related question is the conservation of allotargets in the *Rtl1* mRNA for some of the antisense microRNAs. Are these allotargets conserved because they increase the number of target sites for cleaving the mRNA, because the allotargets ‘sponge’ the microRNAs, or for some more complex process of control (‘information processing’)? Consider the conserved allotarget of miR-136-3p that overlaps the Dicer cleavage site of pre-miR-127 (Fig. 3a). Does miR-136-3p influence the release of miR-127?

The interactions among nuclear and cytoplasmic RNAs, both coding and noncoding, are likely to be complex, and will be a challenge to disentangle experimentally. We agree with Dr. Berezikov that *trans* allotargets of the *Rtl1*-associated microRNAs are likely to be an important part of the evolutionary story. Another layer of complexity, not discussed in our paper, is that sense and antisense *RTL1* transcripts probably are targeted by *trans*-acting microRNAs encoded at other loci. For example, the maternally-expressed *RTL1-antisense* transcript has multiple predicted binding sites for miR-335-5p, a paternally-expressed miRNA that is processed from an intron of the paternally-expressed protein-coding gene *MEST* [58]. Comparative sequence analysis can suggest possible interactions but it will take experimental approaches to sort the wheat from the chaff.

### Reviewer 2: Bernard Crespi

This is a fascinating and important article that describes a detailed hypothesis, with molecular-evolutionary support, for understanding the molecular nature of a genomic imprinting conflict mediated by miRNAs that interact with mRNAs. The proposed conflict mechanism is quite novel, and related to green-beard effects (which are of considerable theoretical interest), and it should also be important for other loci, as well as, potentially, human diseases and disorders that are mediated by alterations to genomic imprinting effects. The authors very nicely integrate the theory with the molecular data, and provide a useful set of relevant figures.

The manuscript can be improved and made more accessible and useful in several ways. First, the authors need to provide more background regarding mechanisms of molecular conflict in imprinted-gene systems, and what the main unresolved questions are in this area of research. This should be done in the Abstract, and in the Introduction. Most readers will not be very familiar with imprinted genes, so the authors need to provide more background in the different ways that imprinted genes act in conflict (or cooperation) with one another, and how these known ways relate to the ways described here (which are rather different). The authors should also discuss polar overdominance and the *callipyge* system, in its relation to *RTL1*, for sheep (and humans), provided that they are of sufficient relevance. Second, there are rather too many Figures in the main text. Some of the less important of them (e.g., 2–6 or 3–6) could be relegated to Supplementary material. The authors do need, however, to add a new figure that shows, as clearly and intuitively as possible, how their hypothesis of conflict works (how it evolved and how it is maintained). The writing in the Results is very dense and highly technical and detailed, and will be a real challenge for most readers - a figure will help considerably to make it penetrable. The authors might also add a Figure that depicted the green beard (red whiskers, etc) effects, which will otherwise be hard for readers to conceptualize. Third, the Conclusions and the Results are not as clearly separable as they could be; conclusions can be quite short and describe the main points and implications, and what should be done next to evaluate the hypothesis further. Fourth, the authors should at least briefly discuss the roles of *RTL1* in human disease, including infertility and cancer, and discuss how their proposed hypothesis may relate to disease susceptibilities and forms.

***Authors’ response.***

We thank Professor Crespi for his kind words. In the Introduction, we have added discussion of polar overdominance and the *callipyge* phenotype of sheep, added references to antagonistic effects of MEGs and PEGs in fetal growth in mice and humans, and added references to the effects of RTL1 and associated microRNAs in infertility and cancer. We have moved four Figures to the supplemental materials. The Results section is indeed dense and technical (it was not an easy section to write). We did our best to write simply about an intrinsically complex system. Figure 1c has been added to help readers keep track of the evolutionary changes discussed. We have moved our discussion of sequence conservation from the Discussion into the Results. We recommend [59] to readers who desire an accessible introduction to greenbeard effects.

## Supplementary information

**Additional file 1: Figure S1.** Conservation of sequences of (a) miR-136 (b) miR-127 (c) miR-433 (d) miR-432 and (e) mir-431. microRNAs (antisense) shown above mRNA sequences (sense); dots represent nucleotide identical to human *RTL1*. *Homo* (human, euarchontoglirean), *Ovies* (sheep, laurasiatherian), *Orycteropus* (aardvark, afrotherian), *Dasypus* (armadillo, xenarthran). **Figure S2.** Self-complementarity of (a) miR-127-3p and (b) miR-433-3p. Predicted hairpin structures of pre-miR-127 and pre-miR-433 were obtained from miRDB [60]. The mature microRNAs processed from pre-miRNA hairpins are indicated by yellow background. miRNA seed sequences indicated in red. **Figure S3**. Alignment of seven 33-nucleotide TRB repeats for *RTL1* genes (sense strand) of diverse eutherian species. Nucleotides in red and blue are complementary to pre-miR-432. Blue sequences are complementary to the seeds of miR-432-5p and miR-432-3p (miR-432-5p target cuccaag occurs twice; miR-432-3p target ccaucca occurs once within pre-miR-432). The TRB repeats are disrupted in mouse and rat *Rtl1* genes (see Additional file 1: Fig. S4). **Figure S4.** Partial sequence of the mouse *RTL1* mRNA showing 12-nucleotide TRA repeats, 66-nucleotide TRB2 repeats, and 24-nucleotide TRC repeats. The TRB2 and TRC repeats have been derived from within the ancestral 33-nucleotide TRB repeats. Sequences in blue are complementary to the seed sequences of miR-432-5p and miR-432-3p. Sequences in red are complementary to the seed sequences of miR-434-5p and miR-434-3p. **Figure S5**. Autotargets and allotargets of miR-434-5p and miR-434-3p in the sequence of mouse *Rtl1* mRNA. These complex relations are a consequence of the 24-nucleotide TRC repeats. **Figure S6.** (a) The pre-miR-432 hairpin: CUUGGAG is the seed of miR-432-5p (appears twice); UGGAUGG is the seed of miR-432-3p; 33-nucleotide periodicity of TRB repeats indicated by successive arrow heads. (b) The pre-miR-434 hairpin: CUCGACU (appears twice) is the seed of miR-434-5p; UUGAACC (appears thrice) is the seed of miR-434-3p; 24-nucleotide periodicity of TRC repeats is indicated by successive arrow heads. The mature microRNAs processed from the hairpins are indicated by yellow. Structures of pre-miRNA hairpins from miRdb [60]. (PDF 3063 kb)

## Data Availability

All data used are from publically available databases including reference genomes in NCBI.
