# Supplementary material for: Retrotransposon gag-like 1 (RTL1) and the molecular evolution of self-targeting imprinted microRNAs
Source: Biol Direct. 2019 Oct 22;14:18. doi: 10.1186/s13062-019-0250-0 (PMC6805670; doi:10.1186/s13062-019-0250-0)
Supplement: Supplementary file 1 — Additional file 1: Figure S1. Conservation of sequences of (a) miR-136 (b) miR-127 (c) miR-433 (d) miR-432 and (e) mir-431. microRNAs (antisense) shown above mRNA sequences (sense); dots represent nucleotide identical to human RTL1. Homo (human, euarchontoglirean), Ovies (sheep, laurasiatherian), Orycteropus (aardvark, afrotherian), Dasypus (armadillo, xenarthran). Figure S2. Self-complementarity of (a) miR-127-3p and (b) miR-433-3p. Predicted hairpin structures of pre-miR-127 and pre-miR-433 were obtained from miRDB [60]. The mature microRNAs processed from pre-miRNA hairpins are indicated by yellow background. miRNA seed sequences indicated in red. Figure S3. Alignment of seven 33-nucleotide TRB repeats for RTL1 genes (sense strand) of diverse eutherian species. Nucleotides in red and blue are complementary to pre-miR-432. Blue sequences are complementary to the seeds of miR-432-5p and miR-432-3p (miR-432-5p target cuccaag occurs twice; miR-432-3p target ccaucca occurs once within pre-miR-432). The TRB repeats are disrupted in mouse and rat Rtl1 genes (see Additional file 1: Fig. S4). Figure S4. Partial sequence of the mouse RTL1 mRNA showing 12-nucleotide TRA repeats, 66-nucleotide TRB2 repeats, and 24-nucleotide TRC repeats. The TRB2 and TRC repeats have been derived from within the ancestral 33-nucleotide TRB repeats. Sequences in blue are complementary to the seed sequences of miR-432-5p and miR-432-3p. Sequences in red are complementary to the seed sequences of miR-434-5p and miR-434-3p. Figure S5. Autotargets and allotargets of miR-434-5p and miR-434-3p in the sequence of mouse Rtl1 mRNA. These complex relations are a consequence of the 24-nucleotide TRC repeats. Figure S6. (a) The pre-miR-432 hairpin: CUUGGAG is the seed of miR-432-5p (appears twice); UGGAUGG is the seed of miR-432-3p; 33-nucleotide periodicity of TRB repeats indicated by successive arrow heads. (b) The pre-miR-434 hairpin: CUCGACU (appears twice) is the seed of miR-434-5p; UUGAACC (appears [file 13062_2019_250_MOESM1_ESM.pdf]

Figure S1. Conservation of sequences of (a) miR-136 (b) miR-127 (c) miR-433 (d) miR-432 and (e) mir-431. microRNAs (antisense) shown above mRNA sequences (sense); dots represent nucleotide identical to human *RTL1*. *Homo* (human, euarchontoglirean), *Ovies* (sheep, laurasiatherian), *Orycteropus* (aardvark, afrotherian), *Dasypus* (armadillo, xenarthran).

Figure S2. Self-complementarity of (a) miR-127-3p and (b) miR-433-3p. Predicted hairpin structures of pre-miR-127 and pre-miR-433 were obtained from miRDB [58]. The mature microRNAs processed from pre-miRNA hairpins are indicated by yellow background. miRNA seed sequences indicated in red.

Figure S3: Alignment of seven 33-nucleotide TRB repeats for *RTL1* genes (sense strand) of diverse eutherian species. Nucleotides in red and blue are complementary to pre-miR-432. Blue sequences are complementary to the seeds of miR-432-5p and miR-432-3p (miR-432-5p target cuccaag occurs twice; miR-432-3p target ccaucca occurs once within pre-miR-432). The TRB repeats are disrupted in mouse and rat *Rtl1* genes (see Figure S4).

Figure S4: Partial sequence of the mouse *RTL1* mRNA showing 12-nucleotide TRA repeats, 66-nucleotide TRB2 repeats, and 24-nucleotide TRC repeats. The TRB2 and TRC repeats have been derived from within the ancestral 33-nucleotide TRB repeats. Sequences in blue are complementary to the seed sequences of miR-432-5p and miR-432-3p. Sequences in red are complementary to the seed sequences of miR-434-5p and miR-434-3p.

Figure S5. Autotargets and allotargets of miR-434-5p and miR-434-3p in the sequence of mouse *Rtl1* mRNA. These complex relations are a consequence of the 24-nucleotide TRC repeats.

Figure S6. (a) The pre-miR-432 hairpin: CUUGGAG is the seed of miR-432-5p (appears twice); UGGAUGG is the seed of miR-432-3p; 33-nucleotide periodicity of TRB repeats indicated by successive arrow heads. (b) The pre-miR-434 hairpin: CUCGACU (appears twice) is the seed of miR-434-5p; UUGAACC (appears thrice) is the seed of miR-434-3p; 24-nucleotide periodicity of TRC repeats is indicated by successive arrow heads.The mature microRNAs processed from the hairpins are indicated by yellow. Structures of pre-miRNA hairpins from miRdb [58].

(S1a)

```
                  miR-136-3p                                    miR-136-5p
     3'-UCUGAGUAAACUCUGCUACUAC-5'            3'-AGGUAGUAGUUUUGUUUACCUCA-5'
        ||||||||||||||||||||||                  |||||||||||||||||||||||
5'-UGAAGACUCAUUUGAGACGAUGAUGGAGCAUAAGAAUCCAUCAUCAAAACAAAUGGAGUCCUC-3'  Homo
   ............................G..................................     Ovies
   ............................G..................................     Orycteropus
   ............................G..G...............................     Dasypus
```

(S1b)

```
                  miR-127-3p                                   miR-127-5p
     3'-UCGGUUCGAGUCUGCCUAGGCU-5'            3'-UAGUCUCGGGAGACUCGAAGUC-5'
        ||||||||||||||||||||||                  ||||||||||||||||||||||
5'-ACCAGCCAAGCUCAGACGGAUCCGAUGAUCUUUCUGAAUCAGAGCCCUCUGAGCUUCAGCAG-3'  Homo
   ..............................................................     Ovies
   ..............................................................     Orycteropus
   ..............................................................     Dasypus
```

(S1c)

```
                miR-433-3p                                                   miR-433-5p
    3'-UGUGGCUCCUCGGGUAGUACUA-5'                                  3'-CUUAUUACUGUCCGAGUGGCAU-5'
       ||||||||||||||||||||||                                        ||||||||||||||||||||||
5'-AGAACACCGAGGAGCCCAUCAUGAUCCUUCUCAACACAGAGGAUCUAGCCUCUCUGAAUAAUGACAGGCUCACCGUACUU-3'  Homo
   .....................................G..........................................     Ovies
   ....................................C...........................................     Orycteropus
   ..................................AGAU..........................................     Dasypus
```

(S1d)

```
                 miR-432-3p                                                miR-432-5p
     3'-UCUGUACCUCCUCGGUAGGUC-5'                               3'-GGUGGGUUACUGGAUGAGGUUCU-5'
        |||||||||||||||||||||                                     |||||||||||||||||||||||
5'-CUCCAAGACAUGGAGGAGCCAUCCAGUGGCCCACGUAAGGAAAUAGAGGAUCCACCCAAUGACCUACUCCAAGACCU-3'  Homo
   .G................................A..G.......A.............................U.     Ovies
   ..................................A.C......................................A.     Orycteropus
   .G...............................U..C.........G.A..........................U.     Dasypus
```

(S1e)

```
                 miR-431-3p                                         miR-431-5p
     3'-UCUUCGGGACGUUCUGCUGGAC-5'                        3'-ACGUACUGCCGGACGUUCUGU-5'
        ||||||||||||||||||||||                              |||||||||||||||||||||
5'-GCGAGAAGCCCUGCAAGACGACCUGCAACGUUACCGUCAGUGUGGCCUGCAUGACGGCCUGCAAGACACCU-3'  Homo
   ......C.........................U......................................     Ovies
   ......C....G...G.G.....................AG.............................C     Orycteropus
```

(S2a)

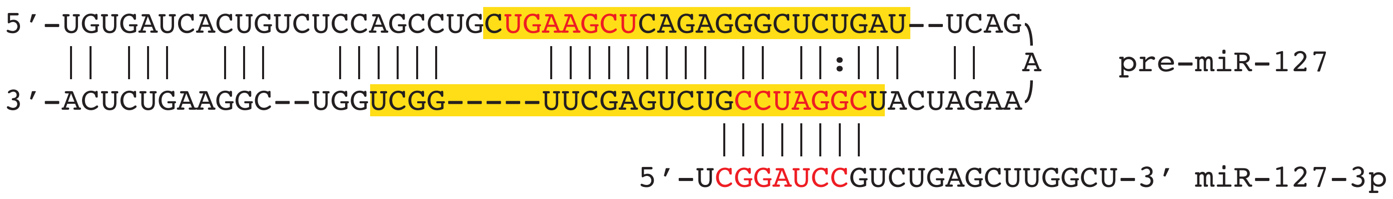
5′-UGUGAUCACUGUCUCCAGCCUGCUGAAGCUCAGAGGGCUCUGAU--UCAG
A
3′-ACUCUGAAGGC--UGGUCGG-----UUCGAGUCUGCCUAGGCUACUAGAA
pre-miR-127
5′-UCGGAUCCGUCUGAGCUUGGCU-3′ miR-127-3p

(S2b)

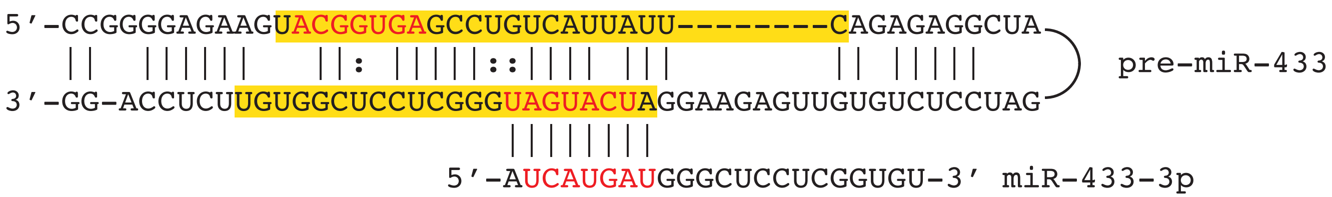
5′-CCGGGGAGAAGUACGGUGAGCCUGUCAUUAUU--------CAGAGAGGCUA
3′-GG-ACCUCUUGUGGCUCCUCGGGUAGUACUAGGAAGAGUUGUGUCUCCUAG
pre-miR-433
5′-AUCAUGAUGGGCUCCUCGGUGU-3′ miR-433-3p

Figure S3

uggagugcggggagaggcagggccagccagcgg-*Homo*
cggagugcaggaggcgaaggagccagccagugg-*Galeopterus*
cggagcgcaggcaggggcgggcccagccagugg-*Nannospalax*
ugcagugcaggagaaggcgggaccagccaaggg-*Heterocephalus*
uggagcgcaggagacggaggggccagcugaggg-*Cavia*
aggcgcgcaggagggggcggggccggcccgcgg-*Oryctolagus*
cgaagcacaggaggug*aggcagcggccccggc-*Ochotona*
cggcagagcggaggcggcggggcuggccg-----*Ovies*
ccaagugcaggaagcgaugggccggccagugg-*Elephantulus*
ccaagugcaggaagcguuggagccagccagugg-*Trichechus*
ccgagcgcaggaagcguuggagccagccagcag-*Orycteropus*
ccaggcgcaggcaggg------------------*Dasypus*

* auggcccaggcagcggccc *Ochotona*

cccagcccaggaaaagaaggagccccccagugg-*Homo*
cccagcccgggaaaaaagagagccacccagugg-*Galeopterus*
cccagcccaggaaagccaggagccaccaagugg-*Nannospalax*
cggagcccaggaaagcaaggagccccccagugg-*Heterocephalus*
cccggcggaggaaagcaaggagcaucccagugg-*Cavia*
cccagcccagggaaggaaggagccacccagggg-*Oryctolagus*
ccuggccccagcaaggaaggagccggucagugg-*Ochotona*
-----------------------------gcgg-*Ovies*
agcggcccaggaaguaagggagcuagccagcgg-*Elephantulus*
cccaucccaggaaagaaaagagccacccagugg-*Trichechus*
cucagcccaggaaagaaaggagccacccagugg-*Orycteropus*
----ccccaggacccccagcagccccccagcga-*Dasypus*

cccacuccaggaaauggaagagcugcccacuga-*Homo*
cucacuccaggaaauggaagagcugcccauuga-*Galeopterus*
cucuguccaggaaaugcaagagcugcccauuga-*Nannospalax*
cucuugccaggaaauagaagagcugccuacuga-*Heterocephalus*
cucccgccaggaaguagaagagcugccuacuga-*Cavia*
cucguuggagaaaauggaagagcugcccacuga-*Oryctolagus*
cccucaggaggagcuggaagagcugcccacuga-*Ochotona*
ccuggcccaggaaacgggagagcaguccauuga-*Ovies*
cccccuccaggaaucggcagcguugcccacuga-*Elephantulus*
cucacuccaggaaauggaagagcugcccauuga-*Trichechus*
cucacuccagggaauggaagagcugcccacuga-*Orycteropus*
ccccuccaggagauggaagugcugcccacuga-*Dasypus*

ucuacuccaagacauggaggagccauccagugg-*Homo*
ucuacuccaagacauggaggagccauccagugg-*Galeopterus*
ucuacuccaagacauggaggagccauccagugg-*Nannospalax*
ucuacuccaagacauggaggagccauccagugg-*Heterocephalus*
ucuacuccaagacauggaggagccauccagugg-*Cavia*
ucuacuccaagacauggaggagccauccagugg-*Oryctolagus*
ucuucuccaagacauggaggagccauccagugg-*Ochotona*
ccuccgccaagacauggaggagccauccagugg-*Ovies*
ucuccuccaagccauggaggagccauccagugg-*Elephantulus*
ucuacuccaagacauggaggagccauccagugg-*Trichechus*
ucuacuccaagacauggaggagccauccagugg-*Orycteropus*
cguccgccaagccauggaggagccauccagugg-*Dasypus*

cccacguaaggaaauagaggauccacccaauga-*Homo*
cccacauaaggaaauagaggauccacccaauga-*Galeopterus*

cccauguaaggaaauagaggauccacccaauga-*Nannospalax*
cccacauagggaaauagaggauccacccaauga-*Heterocephalus*
cccgcauagggaaauagaggauccacccaauga-*Cavia*
cccacguaaggaauuagaggauccacccaauga-*Oryctolagus*
cccacguaaggaaauagaggauccacccaauga-*Ochotona*
cccacauagggaaauaaaggauccacccaauga-*Ovies*
cccacgucaggaaauagaggauccacccaauga-*Elephantulus*
cccacaucaggaaauagaggauccacccaauga-*Trichechus*
cccacaucaggaaauagaggauccacccaauga-*Orycteropus*
cccaugucacgaaauagggaauccacccaauga-*Dasypus*

ccuacuccaagaccuggaggagucaugcaacgg-*Homo*
ccuacuccaagacauggaggagucaugcgaugg-*Galeopterus*
ccuacuccaagacauggaggagucaugcagugg-*Nannospalax*
ccuacuccaagacauggaggagucaugcaaugg-*Heterocephalus*
ccuacuccaagacauggaggagucaugcuaugg-*Cavia*
ccuacuccaagaccuggaggagucaugcaacgg-*Oryctolagus*
ccuacuccaagacguggaggagucaugcaaugg-*Ochotona*
ccuacuccaagacuuggaggagucaugcgaggg-*Ovies*
ccuacuccaagacauggaggagucacacaacgg-*Elephantulus*
ccuacuccaagacauggaggagucaugcaaugg-*Trichechus*
ccuacuccaagacauggaggagucaugccacgg-*Orycteropus*
ccuacuccaagacuuggaggaguca---cacgu-*Dasypus*

uucacaccaggcaagggggauccacucagugg-*Homo*
ugcacgccaggcaauggaggagccacucaauga-*Galeopterus*
uucacaccaggcaaggguggauccacucaacga-*Nannospalax*
uucucaccaggcaauggaggauccacucaauga-*Heterocephalus*
uucugaccaggcaacggaggauccacucccuga-*Cavia*
cucacgccagccaacggggggucuacguagcga-*Oryctolagus*
uucacgcca------gggggauccacgcaauca-*Ochotona*
uucucaucuggaagagggggguccauucggagg-*Ovies*
uucucgccaggaagagggggguccaagcagcga-*Elephantulus*
uucacgccaggaaaugggggauccacgcaauga-*Trichechus*
uucacaccaggagacgggggauccacucaacga-*Orycteropus*
cu---gccugg---ugguggaucccgagcgcgc-*Dasypus*

Figure S4

```
TRA repeats
gagcccaggcgg
gggcccaggcag
gggcccaggcgg
aggcccaggcgg
agacccaggugg
aggcccaggcgg
aggcucaggcug
aagcccagguug
aggcccagguug                         TRB2 repeats
aggcccaggcggggccagccagcggcccagcccaggaaaagaaggagccccccaguggcccacuccaggaaauggaaga
             gcugcccacugaucuacuccaagacauggaggagccauccaguggcccacguaaggaaauagagga
             uccacccaaugaccuacuccaagaccuggaggagucaugcaguggcccccaccaggaaaugcaaga
             gcuacccacugaucuacuccgggaaguggaggagccauccaguggccccuaccaggaaaugcaaga
             gcuacccacugaucuacuccgggaaguggaggagccauccaguggccccuaccaggaaaugcaaga
             gcuacccacugaucuacuccgggaaguggaggagccauccaguggccccuaccaggaaaugcaaga
             gcuacccacugaucuacuccgggaaguggaggagccauccaguggccccuaccaggaaaugcaaga
             gcuacccacugaucuacuccgggaaguggaggagccauccagcgaccccugugagg
                       gaucuaccccaagacauggaggaauca
              agugaugaugguucgaaccagg
            agucgagugaugguucaaaccacg
            aauuaaguaaugguucaaaccaug
            agucgagcuuugguucaaacccag
            agucgagcgauguuuccaaccugg
            agucaagcgguugguucaaaccagg      TRC repeats
            aguccagcgaugguucacaaaagg
            agucaagcuaugauucaaacccgg
            aguuaagugauaauucaaaccagg
            aguuaagcgauaauucgaaccagg
            agucaagcgacaguucaaacc---
            agucaagugauauuucaaaccaggaagggucgga   TRB repeats
             accacucagugaggcauccgauuauagcaugga
```

(Figure S5)

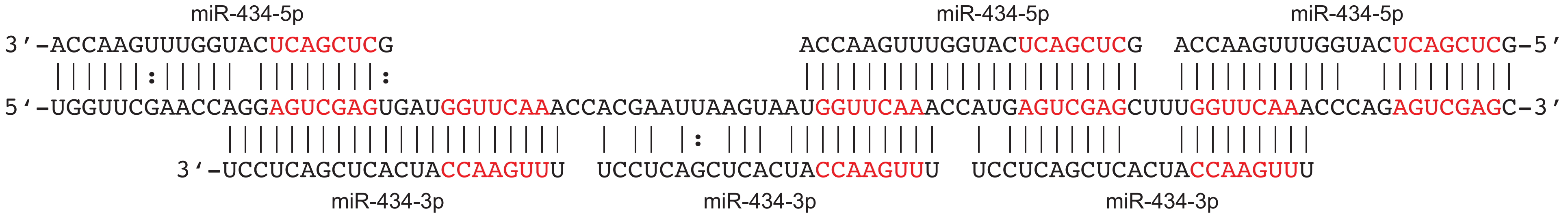
miR-434-5p
3′-ACCAAGUUUGGUACUCAGCUCG
miR-434-5p
ACCAAGUUUGGUACUCAGCUCG
miR-434-5p
ACCAAGUUUGGUACUCAGCUCG-5′
5′-UGGUUCGAACCAGGAGUCGAGUGAUGGUUCAAACCACGAAUUAAGUAAUGGUUCAAACCAUGAGUCGAGCUUUGGUUCAAACCCAGAGUCGAGC-3′
3′-UCCUCAGCUCACUACCAAGUUU
UCCUCAGCUCACUACCAAGUUU
UCCUCAGCUCACUACCAAGUUU
miR-434-3p
miR-434-3p
miR-434-3p

(6a)

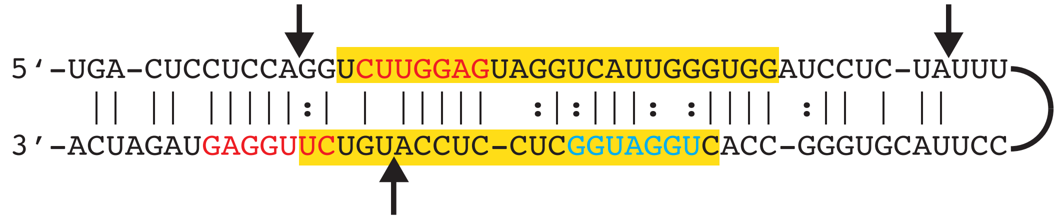
5'-UGA-CUCCUCCAGGUCUUGGAGUAGGUCAUUGGGUGGAUCCUC-UAUUU
3'-ACUAGAUGAGGUUCUGUACCUC-CUCGGUAGGUCACC-GGGUGCAUUCC

(6b)

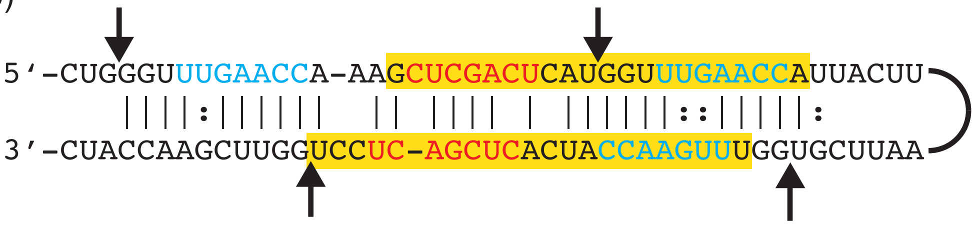
5'-CUGGGUUUGAACCA-AAGCUCGACUCAUGGUUUGAACCAAUUACUU
3'-CUACCAAGCUUGGUCCUC-AGCUCACUACCAAGUUUGGUGCUUAA
